# 

**Published:** 1867-06

**Authors:** 


### THE CHICAGO MEDICAL JOURNAL.

**Edited by J. ADAMS ALLEN, M.D., LL.D.**

Prof. Principles and Practice of Medicine and Clinical Medicine in Rush Med. College.

All letters for the JOURNAL should be addressed to DR. J. ADAMS ALLEN, P.O. Box 1948, Chicago, Ill.

**TERMS---$2 PER ANNUM, IN ADVANCE.**

$3 if not paid before 1st of July in each year. Single copies, 25 cents.

### *To Subscribers.*

In the transfer of names of subscribers from the mail-book, now in the sere and yellow leaf of a quarter of a century, to a new one, it has been found that a number of names were accidentally omitted. This No. is forwarded to the address of every subscriber whose name appears on the books, old or new. Any error will be personally corrected by the editor, on information thereof. Especial attention will be given to correct publication of receipts, not otherwise acknowledged, each month. The editor's private correspondence is already so burdensome that he must positively decline all unnecessary epistolary exercise.

Subscribers are reminded that, after the 1st of July, according to our published rates, *Three Dollars*, instead of *Two Dollars*, is the annual price of the JOURNAL. The low rate at which the JOURNAL is furnished, renders adhesion to the rule of advanced rate imperative.

